# Neutrophils Exert a Suppressive Effect on Th1 Responses to Intracellular Pathogen *Brucella abortus*

**DOI:** 10.1371/journal.ppat.1003167

**Published:** 2013-02-14

**Authors:** Elías Barquero-Calvo, Anna Martirosyan, Diana Ordoñez-Rueda, Vilma Arce-Gorvel, Alejandro Alfaro-Alarcón, Hubert Lepidi, Bernard Malissen, Marie Malissen, Jean-Pierre Gorvel, Edgardo Moreno

**Affiliations:** 1 Programa de Investigación en Enfermedades Tropicales, Escuela de Medicina Veterinaria, Universidad Nacional, Heredia, Costa Rica; 2 Centre d'Immunologie de Marseille-Luminy (CIML), Aix-Marseille University, Marseille, France; 3 Institut National de la Santé et de la Recherche Médicale (INSERM), U1104, Marseille, France; 4 Centre National de la Recherche Scientifique (CNRS), UMR7280, Marseille, France; 5 Departamento de Patología, Escuela de Medicina Veterinaria, Universidad Nacional, Heredia, Costa Rica; 6 Unité de Recherche sur les Maladies Infectieuses et Tropicales Emergentes (URMITE), Marseille, France; 7 Centre National de la Recherche Scientifique, (CNRS), UMR7278, Marseille, France; 8 Institut National de la Santé et de la Recherche Médicale (INSERM), U1095, Marseille, France; 9 Instituto Clodomiro Picado, Facultad de Microbiología, Universidad de Costa Rica, San José, Costa Rica; University of California, Davis, United States of America

## Abstract

Polymorphonuclear neutrophils (PMNs) are the first line of defense against microbial pathogens. In addition to their role in innate immunity, PMNs may also regulate events related to adaptive immunity. To investigate the influence of PMNs in the immune response during chronic bacterial infections, we explored the course of brucellosis in antibody PMN-depleted C57BL/6 mice and in neutropenic mutant Genista mouse model. We demonstrate that at later times of infection, *Brucella abortus* is killed more efficiently in the absence of PMNs than in their presence. The higher bacterial removal was concomitant to the: i) comparatively reduced spleen swelling; ii) augmented infiltration of epithelioid histiocytes corresponding to macrophages/dendritic cells (DCs); iii) higher recruitment of monocytes and monocyte/DCs phenotype; iv) significant activation of B and T lymphocytes, and v) increased levels of INF-γ and negligible levels of IL4 indicating a balance of Th1 over Th2 response. These results reveal that PMNs have an unexpected influence in dampening the immune response against intracellular *Brucella* infection and strengthen the notion that PMNs actively participate in regulatory circuits shaping both innate and adaptive immunity.

## Author Summary

In some diseases the predominant cells recruited are PMNs while in others are mononuclear leukocytes. Traditionally, this marked the difference between acute and chronic infections, a perspective reinforced by *in vivo* models in which immune cells are depleted by means of antibodies. However, these models have several drawbacks and knock-out mice were generated to dissect the functionality of immune cells. Despite this, the study of PMNs in infections in which adaptive immunity plays a role has been precluded by the absence of long-lasting neutropenic models. A mouse strain named Genista, in which the defect is the absence of PMNs has been developed; thus, making possible to explore the role of PMNs during adaptive immunity in chronic infections. We have used *Brucella*, an intracellular pathogen that avoids degranulation and stands the killing action of PMNs. Instead, *Brucella* causes chronicity, inducing granulomas, recruitment of macrophages/DCs and a robust adaptive immune response. We found that the absence PMNs is non-lethal and favors *Brucella* elimination at later times of infection, a phenomenon that correlates with the balance of Th1 over Th2 response. We propose that beside their role in primary bacterial elimination, PMNs can dampen and participate in regulatory circuits of adaptive immune response.

## Introduction

Neutrophils are the first line of defense against microbial pathogens. Upon bacterial infection, these polymorphonuclear leukocytes (PMNs) become activated and are rapidly recruited to the infection site where they can efficiently constrain and kill microbes via phagocytosis, extracellular release of granule contents, cytokine secretion, and the formation of neutrophil extracellular traps [1]. In addition to playing a primary role during the course of innate immunity against acute bacterial infections, PMNs may also influence adaptive immunity [2]–[5]. For instance, PMNs seem to compete with dendritic cells (DCs) and macrophages (Møs) for the availability of antigen [6], shed large membrane vesicles that inhibit the maturation of monocyte-derived DCs and monocyte-derived Møs [7], [8] and are capable of negatively influencing B and CD4^+^ T cell responses activity [6]. The role of PMNs as key regulators of NK cell functions has also been confirmed in patients with severe congenital neutropenia and autoimmune neutropenia [9]. Therefore, in addition to their direct antimicrobial activity, mature neutrophils seem to be endowed with unsuspected immunoregulatory functions that seem to be conserved across species [9].

The short lifespan of PMNs has hampered the assessment of PMN functions during the course of adaptive immunity in long lasting bacterial infections. One model that has been used to evaluate the role of PMNs *in vivo* during bacterial infections has been the early depletion of PMNs by means of pharmacological agents or by antibodies directed to surface protein antigens [10], [11]. Cyclophosphamide or vinblastine PMN depletion has the inconvenience of suppressing myelopoiesis and therefore, negatively affecting the generation of other leukocytes, beside neutrophils [11]. The most valuable *in vivo* model has been the early depletion of mouse PMNs using antibodies directed against Ly6C and Ly6G surface antigens. Although this murine model has been validated with several bacterial pathogens [12]–[17], it has been also questioned. Indeed, in addition to their efficient capability to deplete PMNs, antibodies directed against Ly6C and Ly6G also remove a small subset of inflammatory monocytes and a reduced population of CD8^+^ T cells [10], [15]. Moreover, the long term *in vivo* investigation of chronic bacterial infections in antibody-treated mice is precluded by the fast immune response mounted by the animals against foreign immunoglobulins, which neutralize the PMN depletion effect.

Recently, the generation of a mutant neutropenic mouse strain, named Genista, has been reported [18]. Neutropenia in this mutant animal is the result of a point mutation in the zinc finger protein Growth Factor Independence 1 (Gfi1), which interrupts the maturation of promyelocyte precursors into mature functional PMNs [18]. In contrast to murine strains harboring null alleles of the *Gfi1* gene which display many immune system defects [19], *Genista* mice show normal body weight and viability through time, and a very limited impact in T and B cell function or lymphopoiesis [18]. Genista CD4+ and CD8+ T activated cells display normal rates of proliferation and the mutation has no effect on Th1 and Th2 polarization [18]. Along the block in the terminal stages of granulopoiesis, the *Genista* mutation induces a slight increase of monocytes in the bone marrow and mice display impairment of NK maturation linked to the absence of PMNs [9], [18]. In agreement with PMN antibody depleted mice, *Salmonella*-inoculated Genista mutants also fail to remove the bacterium and die within two or three days, revealing the primary role of PMNs in acute bacterial infections [18].

*Brucella abortus* is an intracellular α-*Proteobacteria* that causes long lasting infections. The chronicity of infection results from the ability of *Brucella* to survive within host cells following phagocytosis by adapting to intracellular conditions that involve thwarting the host's normal immune defenses [20], [21]. One characteristic of *Brucella* is to behave as a stealthy pathogen that circumvents proinflammatory responses [22]. This furtive strategy is related to the negligible activity of those molecules that bear marked pathogen-associated molecular patterns in other bacteria. As consequence *Brucella* triggers low proinflammatory responses at the initial stages of infection, opening an immunological “window” that gives time for the bacterium to reach sheltered intracellular niches before effective Th1 immunity becomes activated [22]–[24].

PMNs are the first cells described to encounter *Brucella* after invasion [25], [26]. However, in contrast to other intracellular Gram negatives such as *Salmonella* pathogens, *Brucella* organisms stand the killing action of PMNs and barely induces degranulation of these cells [27], [28]. As consequence, infected PMNs may serve as “Trojan Horse” for transporting brucellae to regional lymph nodes and thereafter to the entire reticular endothelial system [27], [29]. Moreover, in naïve individuals, *Brucella* does not promote the recruitment of PMNs at the infected site, influence the leukocyte blood counts or induces significant levels of proinflammatory cytokines at the onset of infection (before 48 h), a property that is consistent with the low stimulatory action of *Brucella* pathogen-associated molecular patterns, including its lipopolysaccharide, lipoproteins, ornithine-containing lipids and flagellum [22]–[24], [30].

In order to further investigate the influence of PMNs during the adaptive immune response against long-lasting bacterial infections, we have used the neutropenic Genista model to explore the course of brucellosis. We have evaluated the cellular immune response at the acute and chronic brucellosis phases, respectively. The absence of PMNs in *B. abortus* infected Genista mice promoted a higher activation of CD4^+^ and CD8^+^ T and B cells and a stronger recruitment of monocytes, which resulted in a decrease of bacterial spleen loads. This reveals that PMNs have an unexpected influence in dampening the adaptive immune response against *Brucella* infection by down-modulating features of the adaptive immunity.

## Results

In order to determine the influence of murine PMNs in the immune response during *B. abortus* infection, a time course protocol was designed to include: neutrophil depletion, bacterial inoculation, spleen bacterial counts, histopathological examination and analysis of immune cells (Figure S1). As expected, at the second week of infection mice depleted of PMNs with anti-RB6 (PMN-depleted) already mounted an efficient response against the foreign monoclonal antibody, neutralizing the PMN depletion effect (Figure S1). In spite of this, experiments in PMN-depleted mice were carried out until 15 days to follow the outcome of the infection after the initial PMN depletion.

### PMNs are recruited in the spleen of infected wild type (WT) mice at early times of the acute *Brucella* infection

**Figure 1:**
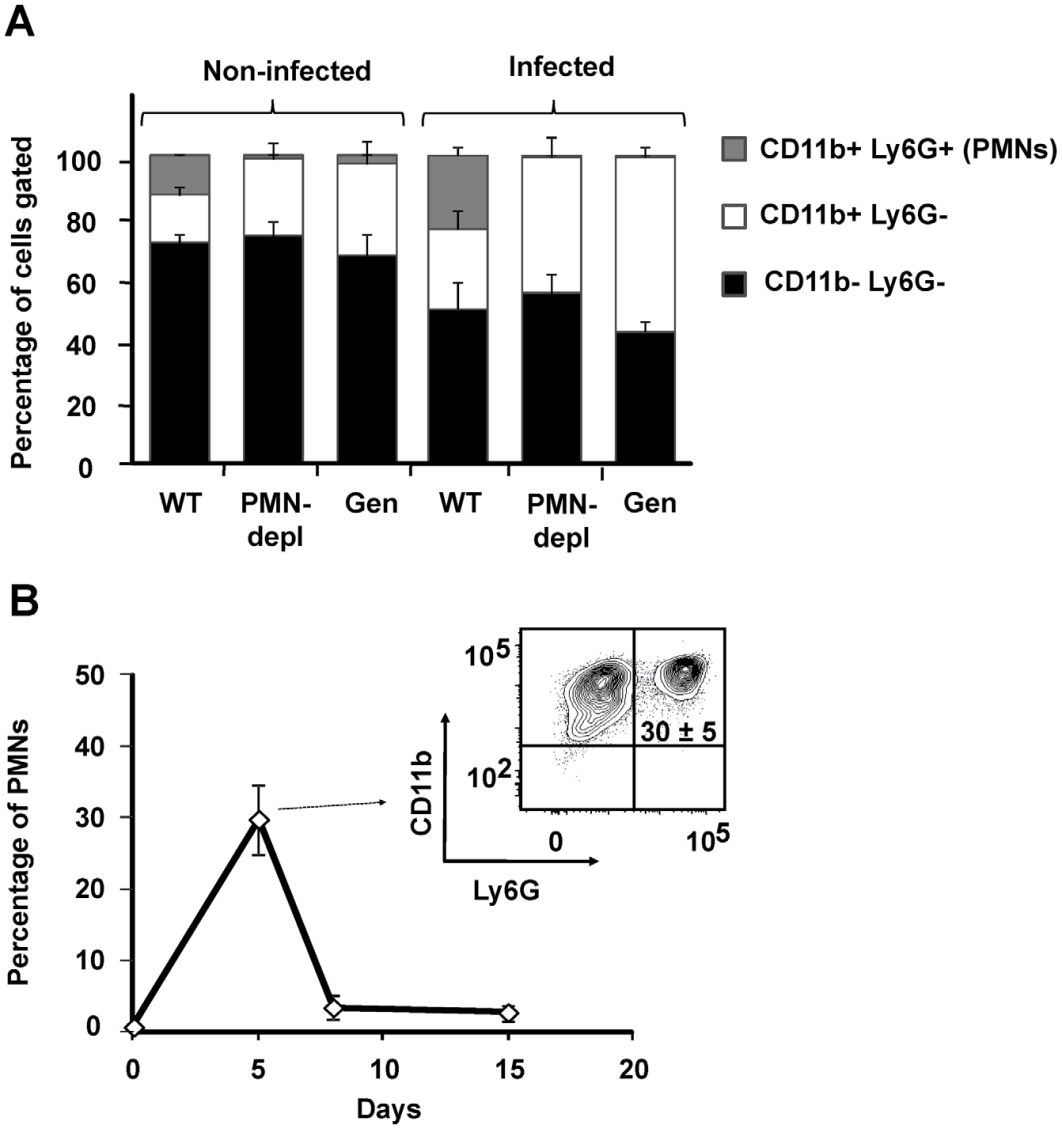
PMNs are recruited at 5 days post-infection in WT. (A) Blood leukocytes from infected and non-infected WT, PMN-depleted (after 5 days of PMN depletion) and Genista mice stained with anti-Ly6G and anti-CD11b were analyzed by flow cytometry. (B) Splenocytes from infected WT mice stained with anti-Ly6G and anti-CD11b at 5, 8 and 15 days post-infection were analyzed by flow cytometry.

It has been demonstrated that *Brucella* barely influences the leukocyte blood counts and does not recruits PMNs in the spleen (the main target organ for *B. abortus* replication) during the first 48 h, a time lapse that corresponds to the onset of infection [22], [30], [31]. Therefore, it was relevant to determine the proportion of PMNs in blood and spleen after the onset of *B. abortus* infection, during the course of the acute phase [32]. While at 5 days post-infection WT mice showed a twofold change in the proportion of blood PMNs, Genista and PMN-depleted mice did not demonstrate circulating PMNs (Figure 1A). Nevertheless, in Genista and PMN-depleted mice a significant increase in the proportion of other blood leukocytes (CD11b+/Ly6G−) was observed (Figure 1A). At 5 days post-infection the relative number of PMNs increased in spleen of WT animals (Figure 1B), indicating that these granulocytes are recruited in this organ at early times of the acute infection phase. PMNs already decrease after eight and fifteen days of infection in C57BL/6 mice (Figure 1B) and no increase in PMN was observed afterwards (not shown), following what has been observed in human patients and infected animals [33], [34]. As expected, a dramatic reduction of PMNs was observed in the spleens of Genista and PMN-depleted mice by histological examination or flow cytometry (not shown).

### Genista mice eliminate *B. abortus* more efficiently than WT counterparts at the chronic infection phase

**Figure 2:**
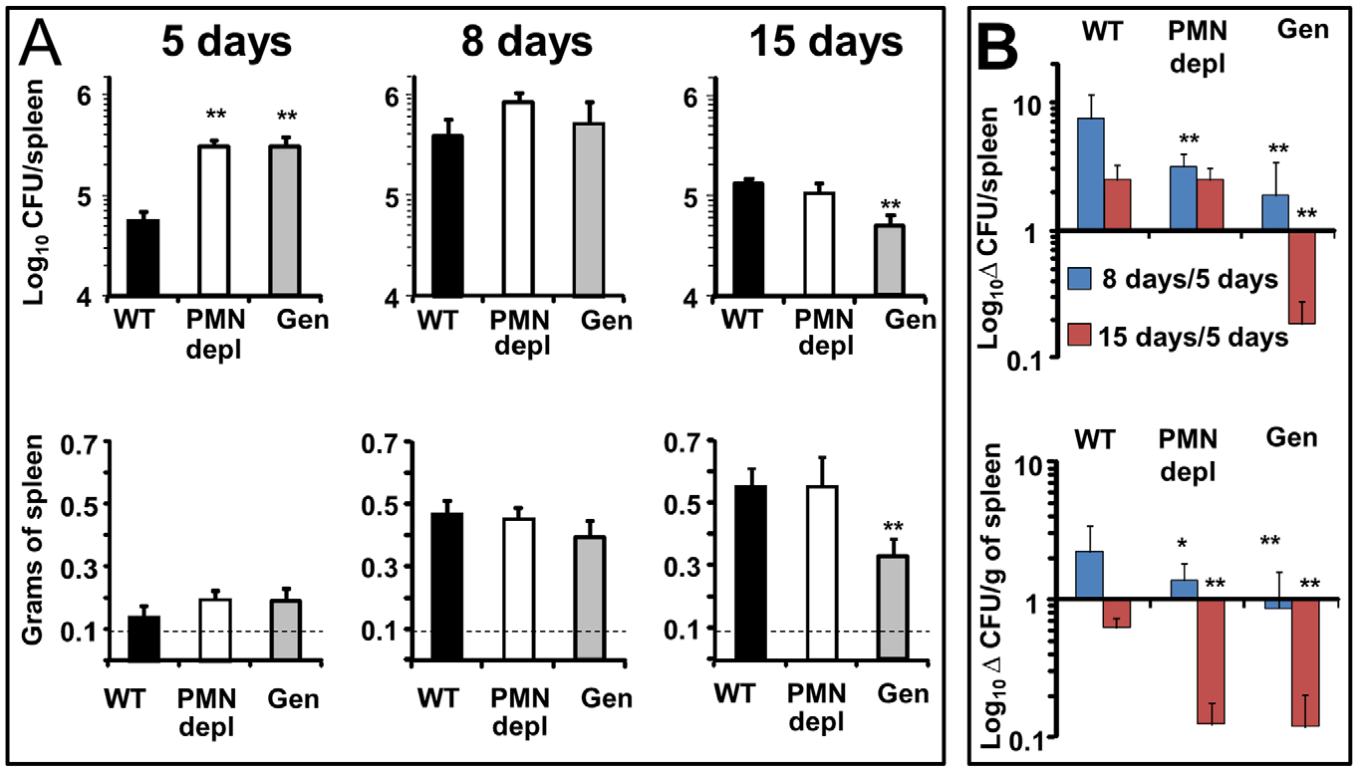
Bacterial loads, spleen weights and rate of change in CFUs per spleen and CFUs per spleen weight over time. (A) Mice were infected i.p. with 10^6^ CFUs, at indicated times CFU/spleen and spleen weights were determined. Background levels of PBS injected mice in each period (dashed line) are depicted. (B) Rate of change in CFU/spleen (Δ CFU/spleen) and rate of change in CFU/spleen weight (Δ CFU/grams of spleen) were calculated over time using the following equations: Δ CFU/spleen = mean CFUs 8 or 15 days/CFUs 5 days ± SD; Δ CFU/grams of spleen = CFU/grams of spleen 8 or 15 days/5 days ± SD. Values of p<0.05 (*) and p<0.01 (**) in relation to WT values are indicated above the bars. Experiment was repeated three times with similar results.

*Brucella* replication in the spleen of mice becomes prominent throughout the first and second week, during the acute phase of infection [32]. At this time, the predominant Th1 adaptive immune response against *B. abortus* is established in the mouse model [32]. At 5 days post-infection, PMN-depleted and Genista mice showed an increase in the spleen bacterial loads as compared to WT counterparts (Figure 2A). The early increase of *Brucella* loads in the spleen was not due to higher bacterial colonization of this organ in neutropenic mice. Indeed, after 16 h of *Brucella* infection the CFU/spleen and spleen weights displayed similar values in the controls and PMN-depleted mice (Figure S2). This indicates that PMNs play an important role in influencing the early replication of *Brucella* in this organ in C57BL/6 mice. At 8 days post-infection, bacterial loads in all three groups had reached similar numbers (Figure 2A), demonstrating that neutropenic mice are able to circumvent the initial increase of *Brucella* replication in the spleen (Figure 2B). Strikingly, at 15 days post-infection corresponding to the beginning of the chronic phase, Genista mice showed a significant decrease in the number of CFUs per spleen in comparison to WT mice (Figure 2A). This reduction in the number of CFUs in the Genista mice was even more significant at 21 days of infection (Figure S3). Concomitantly to the rising of PMNs after the first week of depletion (Figure S1), the anti RB6-8C5 treated mice displayed similar spleen bacterial counts as the controls (Figure 2A). However, taking into consideration that the burden of *Brucella* present in the spleen at 5 days of infection was significantly higher in PMN deficient mice, then, the absolute bacterial elimination at 15 days of infection is highly significant in both mice, being more conspicuous in the Genista mice (Figure 2B).

All three groups of mice displayed a similar increase in spleen weights at 5 and 8 days post-infection (Figure 2A). However, at 15 days post-infection the spleen weight of WT and PMN-depleted mice significantly increased in relation to 8 days post infection. In contrast, in the Genista mice the spleen weight did not increase in relation to 8 days post-infection, a fact that correlates with the even lower number of CFUs in these mice.

**Figure 3:**
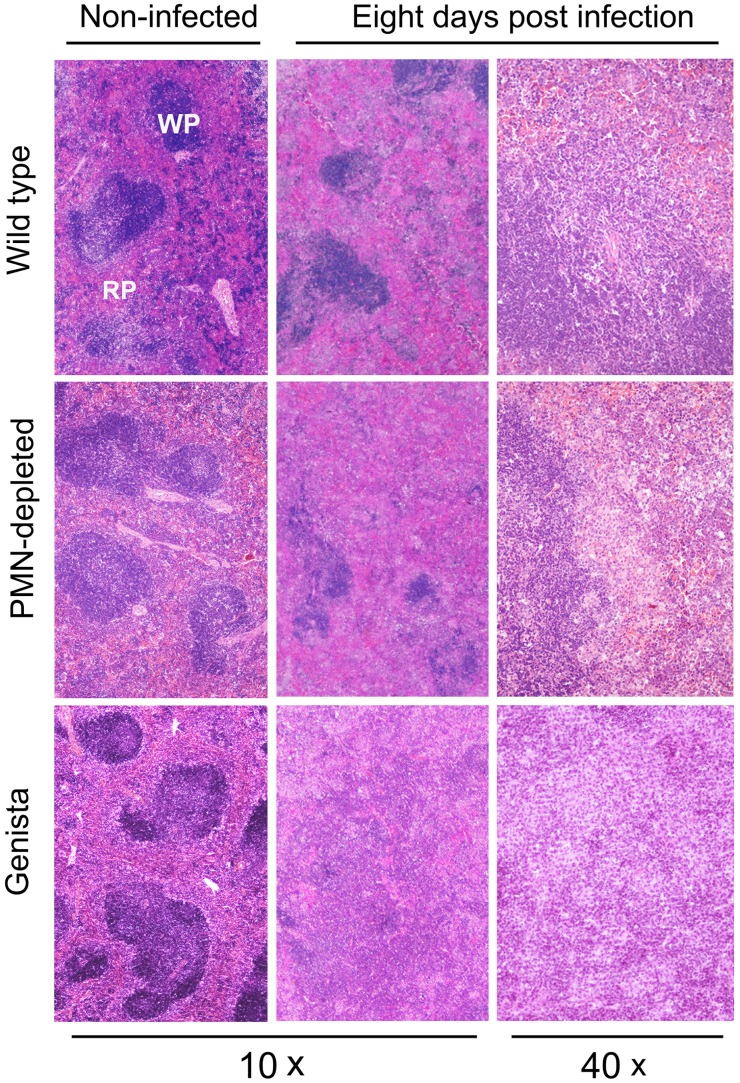
Lymphoid depletion, macrophage infiltration and granuloma formation becomes more prominent in spleens of PMN-depleted and Genista than in WT *Brucella* infected mice. Spleens from infected (10^6^ CFUs) and PBS-treated mice were fixed and stained with hematoxylin and eosin stain. The absence of PMNs in Genista or PMN-depleted non-infected mice did not affect the normal distribution of white pulp (WP) and red pulp (RP) or the overall histological structure of the spleen.

The absence of PMNs in Genista or PMN-depleted uninfected mice did not affect the normal distribution of white and red pulp or the overall histological structure of the spleen (Figure 3). At 8 days post-infection, the augmented spleen size of WT mice displayed a mild lymphoid depletion with moderate macrophage infiltration in the splenic nodules and few neutrophils, multifocal granulomas and some extramedullary hematopoiesis (Figures 3 and S4). It is well known that such granuloma contain the majority of bacteria and bacterial antigen [31], [32] and is mainly composed of cells expressing CD11b, F4/80, MHC-II, which are specific of activated monocytes/Møs. A fraction of these granuloma cells also expresses CD11c and are similar to inflammatory DCs [31], [32]. With the exception of PMNs presence, this general picture is maintained in the PMN-depleted mice. However, in these animals lymphoid depletion, Møs infiltration and granuloma formation becomes more prominent than in WT spleens (Figures 3 and S4). At this time, the histopathological image of the infected Genista spleens is even more conspicuous, with an almost complete disorganization of splenic nodules and the presence of extensive multifocal inflammation and extensive fusion of granulomas (Figures 3 and S4). After 15 days of infection the disorganization of the lymphatic nodules in the spleens of WT, PMN-depleted and Genista mice remains, but with less severity. However, the spleens of Genista mice display more diffuse epithelioid histiocytes infiltration, lower number of discrete granulomas, reduced extramedullary hematopoiesis and less hyperemia than the spleens of WT mice, suggesting an earlier remission (not shown). Altogether these experiments demonstrate that the chronic absence of PMNs favors the elimination of *B. abortus* at later time points, when the Th1 adaptive immune response against *Brucella* becomes evident in mice [32], and that this reduction in bacterial loads correlate with recruitment of macrophagic cells in the spleen.

### B and T lymphocytes are activated and monocytes and mono/DCs phenotype are recruited in neutropenic infected mice

**Figure 4:**
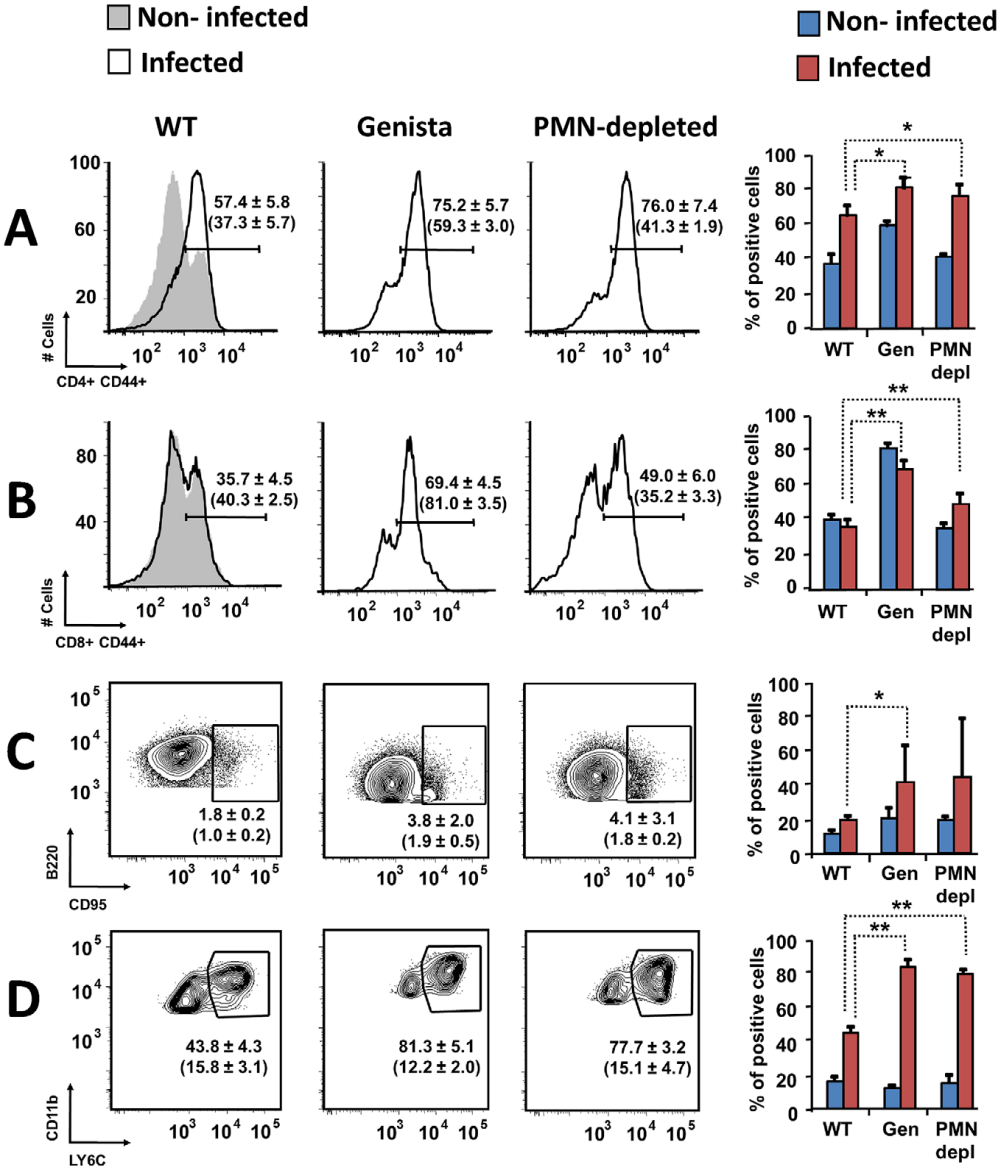
Activation of T and B lymphocytes and recruitment of monocytes in WT and neutropenic mice (Genista and PMN-depleted) at 8 days post-infection. Leukocytes from lymph nodes, were analyzed at 8 days post-infection using (A) CD4^+^/CD44^+^, (B) CD8^+^/CD44^+^, (C) B220^+^/CD95^+^ and (D) CD11b^+^/Ly6C^+^ cell markers. Numbers in parenthesis indicate the percentages of non-infected mice. The percentages of cells found in each of the specified gates are indicated. Values of p<0.05 (*), p<0.01 (**) are indicated.

**Figure 5:**
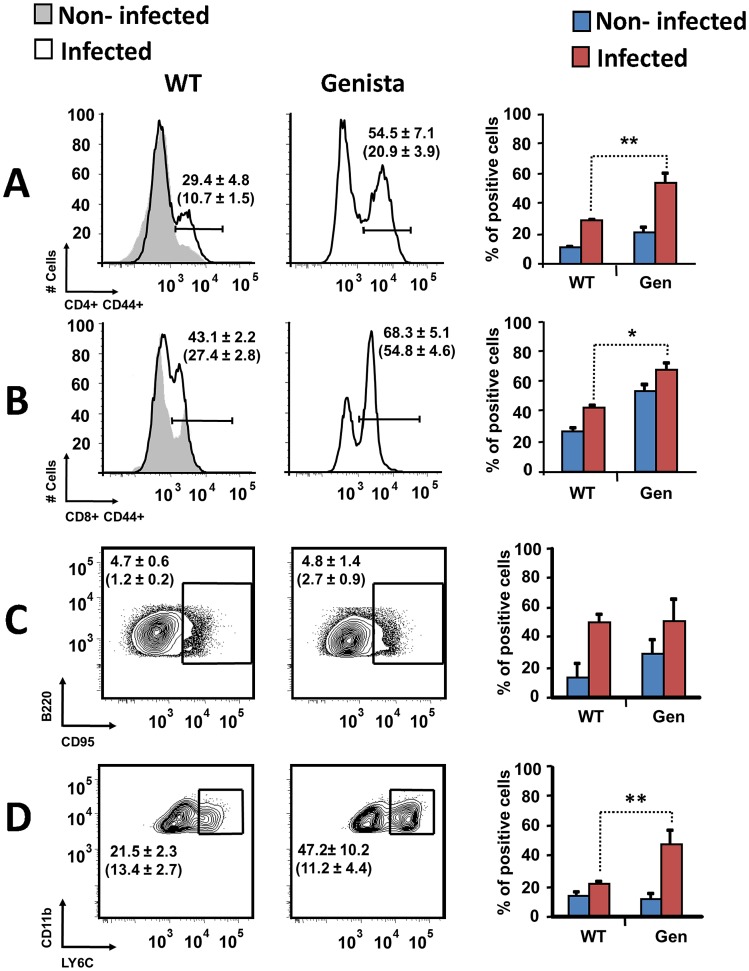
Activation of T and B lymphocytes and recruitment of monocytes in WT and Genista mice at 15 days post-infection. Leukocytes from lymph nodes were analyzed at 15 days post-infection using (A) CD4^+^/CD44^+^, (B) CD8^+^/CD44^+^, (C) B220^+^/CD95^+^ and (D) CD11b^+^/Ly6C^+^ cell markers. Numbers in parenthesis indicate the percentages of non-infected mice. The percentages of cells found in each of the specified gates are indicated. Values of p<0.05 (*), p<0.01 (**) are indicated.

**Figure 6:**
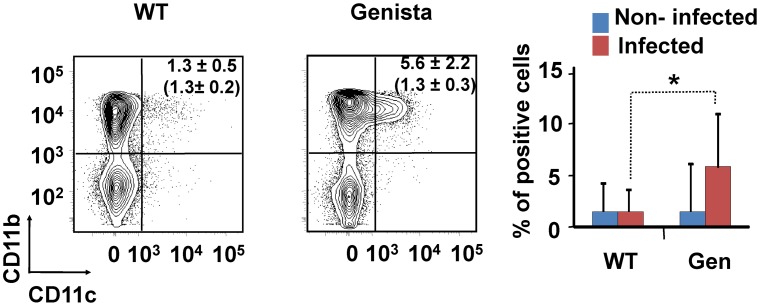
Recruitment of monocytes/DCs (CD11b+/CD11c+) in the blood at 15 days post-infection. Leukocytes from blood were analyzed after 15 days of infection using CD11b^+^/CD11c^+^ cell markers. Numbers in parenthesis indicate the percentages of non-infected mice. The percentages of cells found in each of the specified gates are indicated. Values of p<0.05 (*), p<0.01 (**) are indicated.

Both T and B lymphocytes play a major role in the protective adaptive immune response in experimental murine brucellosis [32]. In the absence of PMNs, at 8 days of infection, there is an increase in the proportion of other leukocytes such as CD4^+^ and CD8^+^ T cells with up-regulated expression of the effector/memory-like activation surface marker glycoprotein CD44 in *Brucella* infected mice (Figures 4A and B). Non-infected Genista mice already had an augmented proportion of T cells expressing CD44 (Figures 4A and B, and Table 1) and displayed a modest but significant increase in the proportion of activated B cells (Figure 4C and Table 1). While CD4^+^ and CD8^+^ T cells still show an up-regulation of CD44 at 15 days post-infection, no differences were observed in B cells at this time point in Genista mice (Figures 5A, B and C). Although some variation was observed between blood, spleen and lymph nodes, the activation and recruitment of these cell types show a common trend (Table S1 and S2). Indeed, the proportion of PMNs and effector/memory CD8+T cells (CD8+/CD44 high) remained grossly within the limits of uninfected spleens in all mice. In contrast, effector/memory CD4+ T (CD4+/CD44 high), activated B cells (B220+/CD95+) and inflammatory monocytes (CD11b+ Ly-6C+) have significantly increased at this time period in the spleen of all mice (Table S1). However, a clearer increase in monocytes occurred in neutropenic animals in relation to WT *B. abortus* infected mice (Figures 4D and 5D), a phenomenon in tune with the inflammatory outcome observed in the spleen (Figure 3). Monocytes significantly augmented in all organs analyzed (Tables 1, S1, and S2). Similarly, at 15 days post-infection (corresponding to the time the number of *Brucella* decreases in the spleen of Genista mice), there is concomitant and significant increase of CD11b^+^/CD11c^+^ mono/DCs phenotype (Figure 6). This population was not observed in spleens or lymph nodes at 15 days post-infection or at earlier time points during the infection process. No changes in other cell populations such as NK cells were observed in the various organs studied (not shown). It has been described that the function, number and distribution of lymphocytes and DCs are within normal ranges in Genista mice [18]. Concomitantly, it seems that all these activation and recruitment events are linked to the ability of neutropenic mice to mount a faster and more efficient adaptive immune response against *Brucella*. Since these cellular events become more conspicuous during the acute and chronic phases of brucellosis, the overall conclusion is that the absence of PMNs influences adaptive immunity.

**Table 1:** Leukocytes in lymph nodes from infected and non-infected WT, PMN-depleted and Genista mice were analyzed by flow cytometry at 8 and 15 days of infection using CD4+/CD44+, CD8+/CD44+, B220+/CD95+, and CD11b+/Ly6C+ cell markers.

		Mice					
Time	Cell markers	WT		Genista		PMN-depleted	
		Non-Infected	Infected	Non-Infected	Infected	Non-Infected	Infected
**8 days**	CD4+/CD44+	37.3±5.7	65.4±5.8	59.3±3.0	75.2±5.7	41.3±1.9	76.0±7.4
	CD8+/CD44+	40.3±2.5	35.7±4.5	81.0±3.5	69.4±4.5	35.2±3.3	49.0±6.0
	B220+/CD95+	1.0±0.2	1.8±0.2	1.9±0.5	3.8±0.8	1.8±0.2	4.1±3.1
	CD11b+/Ly6C+	15.8±3.1	43.8±4.3	12.2±2.0	81.3±5.1	15.1±4.7	77.7±3.2
**15 days**	CD4+/CD44+	10.7±1.5	29.4±4.8	20.9±3.9	54.5±7.1		
	CD8+/CD44+	27.4±2.8	43.1±2.2	54.8±4.6	68.3±5.1		
	B220+/CD95+	1.2±0.2	4.7±0.6	2.7±0.9	4.8±1.4		
	CD11b+/Ly6C+	13.4±2.7	21.5±2.3	11.2±4.4	47.2±10.2		

The percentages of cells found in each of the specified gates are indicated.

### Proinflammatory cytokines in *Brucella* infected neutropenic mice indicate a balance of Th1 over Th2 response

**Figure 7:**
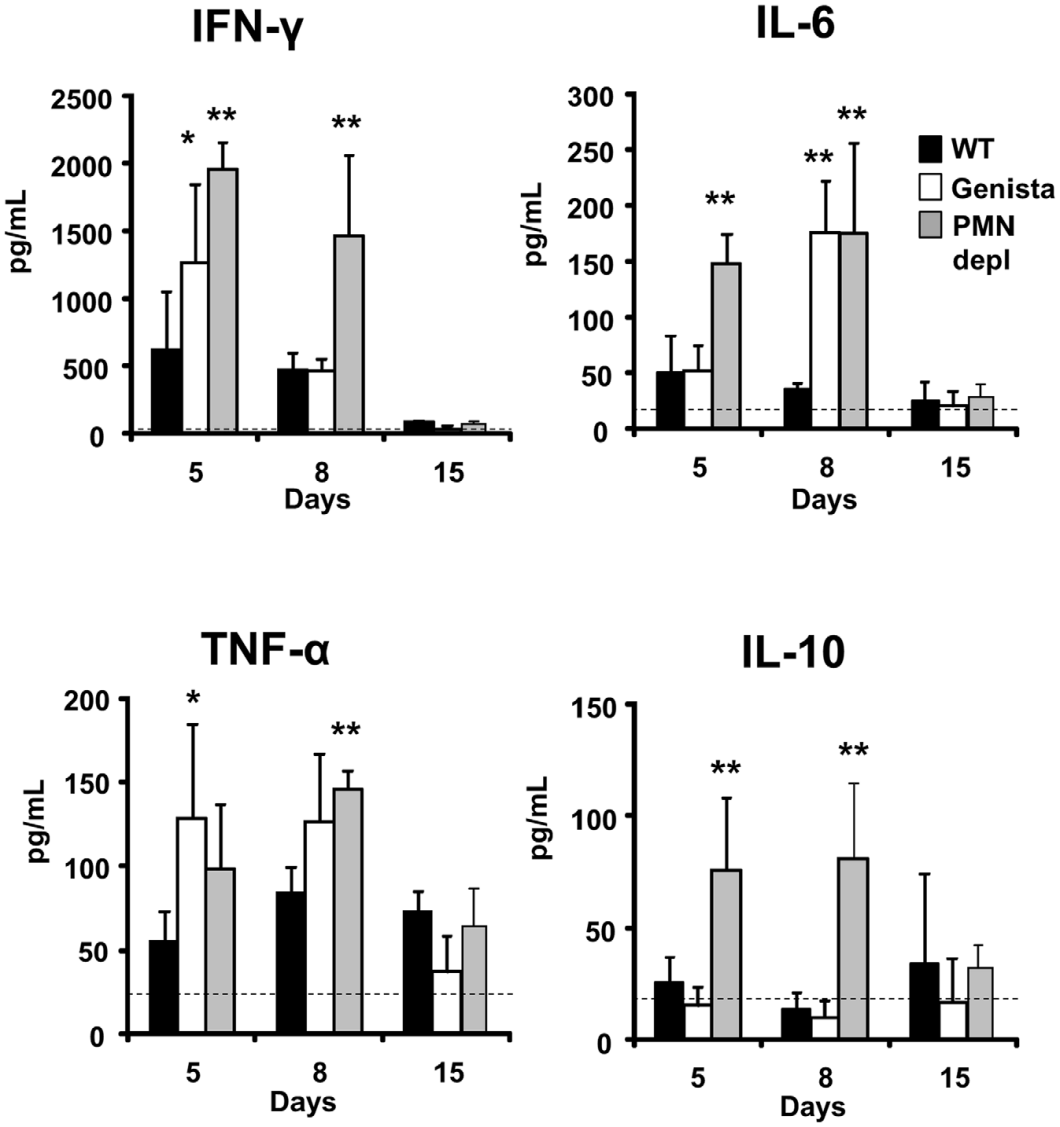
Levels of cytokines detected over time. The levels of INF-γ, IL-6, TNF-α and IL-10 were determined in the sera of WT, PMN-depleted and Genista mice i.p. infected with 10^6^ CFUs of *B. abortus* 2308 at 5, 8 and 15 days post-infection. Background levels of PBS injected mice (dashed line) are depicted in each graphic. Values of p<0.05 (*), p<0.01 (**) are indicated.

The kinetics and quantities of cytokines observed in the infected WT mice are consistent with those described in previous publications [35]–[38]. During the first week of the acute phase, significant amounts of INF-γ and low levels of IL-4 are produced in *B. abortus* infected mice, an event that has been linked to a predominant Th1 immune response during brucellosis. Although there are time differences between the Genista and PMN-depleted mice at 5 and 8 days post-infection, there was a clear tendency in these neutropenic animals in generating larger amounts (up to 2000 pg/mL) of INF-γ at earlier times (Figure 7). This increase of INF-γ in neutropenic mice does not seem to be linked to the augmented number of bacteria in the spleen of these mice at 5 days of infection. Indeed, as observed in Figure S5A, within ranges from 5×10^4^–10^7^ CFU/spleen (corresponding to i.p. bacterial doses ranging from 10^3^–5×10^6^) the amounts of INF-γ barely change their levels, at five days of infection. Only when overloading doses of *Brucella* are used (>5×10^6^), then the differences in INF-γ production become significant in relation with the lower doses (e.g. <10^3^ CFUs). However, in spite of the high numbers of bacteria (about 10^7^ CFU/spleen), the amounts of INF-γ are far below those observed in the neutropenic mice (about ten time less). Following this, the inflammation of the spleen remains within the same limits at ranges of 5×10^4^–10^7^ CFU/spleen (Figure S5B). We have demonstrated previously that, unless overwhelming doses of *Brucella* are injected (>10^8^ CFUs), TNF-α, IL-6 and IL/-10 cytokines seldom reach significant levels in serum at early times of infection [22]. Despite of this, a modest (below 250 pg/mL) but significant increase in IL-6 and TNF-α was observed (Figure 7). The increase of TNF-α has been previously reported in *Mycobacterium* infected PMN-depleted mice [2], suggesting an activation of Mø and DCs. Augmented quantities of IL-10 in *Mycobacterium* infected PMN-depleted mice were also reported [2], but in much larger quantities than those observed here. In agreement with the Th1 predominant immune response during brucellosis, practically no IL-4 or IL-17 cytokines were measurable during *Brucella* infection in either mouse group (Figure S6).

Null mutation in the Gfi-1 gene can act inside T cells and affects the Th1/Th2 balance [39]. In contrast, in Genista mice this mutation has not a direct intrinsic effect on T cells and thus, it does not directly influence the Th1/Th2 balance [18]. Therefore, the Th1 bias observed in *Brucella* Genista infected mice is not the result of the fact that T cells expressed a hypomorphic/partial loss of function mutation of Gfi-1, but rather the consequence of PMNs absence.

## Discussion

PMNs have been viewed as the most efficient effectors cells involved in acute inflammatory responses against bacterial infections. This perspective has been reinforced by the use of antibody-neutropenic mouse models in which bacteria such as *Streptococcus pneumoniae*, *Acinetobacter baumannii*, *Salmonella enterica*, *Listeria monocytogenes*, *Yersinia enterocolitica*, *Staphylococcus aureus*, *Ochrobactrum anthrophi* and *Legionella pneumophila,* among others were used as infecting agents, mostly with fatal consequences [12]–[17], [22], [23]. In addition, lethality due to increased proinflammatory responses mediated by PMNs has also been resolved in antibody-neutropenic models. For instance, it has been demonstrated that in the absence of PMNs, mice infected by the intranasal route with *S. pneumoniae* serotype 8 survived for longer periods than uninfected mice [40] indicating that local proinflammatory responses induced by PMNs may be also lethal. However, due to the fact that PMN antibody depletion cannot be maintained beyond one week, the assessment of the role of PMNs in adaptive immunity in chronic bacterial infections such as brucellosis or tuberculosis has been precluded. Therefore, Genista, a viable neutropenic mouse emerged as an important model to study the influence of PMNs in the development of adaptive immunity [18].

As in other bacterial infections, the absence of PMNs favored the initial increase of bacterial numbers at early times of the acute phase in both neutropenic models [18], [22]. In spite of this, the *B. abortus* higher replication was not lethal in either mouse model. This phenomenon is in clear contrast to what has been recorded in other Gram negative intracellular bacterial infections, such as *Salmonella* or with opportunistic pathogens closely related to *Brucella,* such as *Ochrobactrum* [18], [22], [23]. As the infection progressed, the spleen *Brucella* loads leveled up in both models of neutropenic mice, indicating that PMNs were necessary at early but not at later phases of the acute infection. Strikingly, at the beginning and later on of the chronic phase the number of bacterial CFUs in Genista mice was significantly lower than those detected in WT counterparts. This revealed that the absence of functional PMNs favored bacterial removal at the chronic phase. The higher elimination of *B. abortus* in neutropenic mice correlated to the: i) comparatively reduced spleen swelling; ii) augmented infiltration of epithelioid histiocytes depicted as Mø/DCs; iii) significant recruitment of monocytes and mono/DCs phenotype; iv) higher activation of B and T lymphocytes, and v) increased levels of INF-γ and negligible levels of IL4 indicating a balance of Th1 over Th2 response. Altogether these results indicate that PMNs constrain and regulate the activation of adaptive immune response against intracellular *B. abortus* infection.

In agreement with this proposal, it has been shown that in PMN antibody-depleted mice, *Mycobacterium* loads increased at initial stages of infection but decreased at later time points [2]. Similarly, in the absence of PMNs, inflammatory monocytes are readily recruited at the infection site [18], [41] and express a strong proinflamatory signature against long lasting intracellular bacteria such as *Mycobacterium* [2]. It is known that monocytes play a significant role in replenishing resident macrophages and DCs under normal as well as during inflammatory conditions [42], [43]. However, in contrast to our finding the decrease in *Mycobacterium* loads in PMN-depleted mice was related to augmented levels of the regulatory cytokine IL-10 [2], rather than the higher levels of INF-γ observed during *Brucella* infections. It is relevant to notice that INF-γ has been pointed as the central cytokine in the *in vivo* control and elimination of *Brucella* [37], [38], [44], [45].

Although the mechanisms by which PMNs influence adaptive immunity are not known, the regulatory events exerted by these short living cells may be through direct action on lymphocytes, DCs/Mø and/or via effectors intersecting regulatory circuits of the immune response. For instance, it has been described that activated PMNs inhibit T cell functions and suppress cytokine production through the generation of hydrogen peroxide in advanced cancer patients [46]. In PMNs antibody-depleted mice inoculated with adjuvants, neutrophils suppress the activation of B and CD4+ T cells but not CD8^+^ T cell responses [6]. PMNs have been found to interfere with the ability of DCs and Mø to present antigens shortly after their migration into lymph nodes, probably by competing with the antigen-presenting cells for the available antigen [6]. *Mycobacterium tuberculosis* infections inhibit PMN apoptosis, leading to delayed activation of naïve CD4^+^ T cells [47]. Emerging evidence indicates that PMNs may communicate with T cells through direct cell contact via class I and class II major histocompatibility complex proteins [48], [49] as well as co-stimulatory molecules [50], [51]. Additionally, the ability of PMNs to synthesize and release immunoregulatory cytokines [52] or other molecules may have significant impact on the adaptive response to infection. For example, ectosomes released by human PMNs inhibit the maturation of both monocyte-derived DCs [7] and monocyte-derived Mø [8], possibly by increasing their production of the regulatory TGFβ1 cytokine [7], [8].

Given the recently broad functions assigned to PMNs, it is not surprising that these cells will emerge as important players in regulatory circuits in the innate and adaptive immune systems and in the pathogenesis of numerous disorders, including infection caused by intracellular bacteria [4]. From this and previous experiments [20], [22], [23], [30], it has been demonstrated that the global activity of PMNs in brucellosis is a balance between the positive (direct bacterial elimination) and negative (down-regulation) immune properties. At the onset of infection (the first 48 h) *Brucella* behaves as stealthy pathogen avoiding recruitment of PMNs at the inoculation site and stands the killing action of these cells [20], [22], [23], [30]. At five days post-infection, the presence of PMNs allows the partial elimination of *Brucella* (Figure 2). However, thereafter the presence of PMNs seems detrimental for mounting an efficient adaptive immune response against brucellosis. The activation of T and B cells concomitantly to the recruitment of monocytes and mono/DCs phenotype and to the increase of the cytokines that are central in the elimination of *Brucella* [38], [53], are all augmented in the neutropenic mice. This agrees with the notion that PMNs actively participate in regulatory circuits of the innate and adaptive immune systems. Within this context, it is important to dissect the mechanisms by which PMNs regulate elements of adaptive immunity and to reconsider this short living cells as valuable targets in long lasting chronic infectious as well as non-infectious inflammatory diseases [4].

## Materials and Methods

### Mice strains and infection protocols

C57BL/6 mice were purchased from Charles River Laboratories (L'Arbresle, France), housed under specific pathogen free conditions and handled in accordance with French and European guidelines. The characteristics, source and maintenance of Genista mice have been previously described [9], [18]. Mice were depleted of neutrophils and infected as previously described [22]. Briefly, C57BL/6 mice were depleted of PMNs by means of i.p. injection of 100 mg of RB6-8C5 monoclonal antibody against murine granulocytes in 0.1 mL PBS, 24 h before infection. Then, WT, PMN-depleted and Genista mice were i.p. infected with 0.1 mL of PBS containing 10^6^ CFUs of virulent *Brucella abortus* 2308 obtained from 16 h bacterial tryptic soy broth cultures incubated at 37°C. In one experiment doses of *Brucella abortus* 2308 ranging from 10^3^–10^7^ CFUs were used (Figure S5). Thereafter, the PMN-depleted mice were i.p. injected with 100 mg of RB6-8C5 antibody every three days until sacrificed. Controls were injected with 0.1 mL sterile PBS at the same time. PMN depletion was confirmed by the absence of CD11b+ Ly6G+ cells by flow cytometry and microscopic examination of blood smears and histological sections. PMN depletion lasted at least for seven days. After this time the number of PMNs rapidly increased, and mice become resilient to depletion (Figure S1). A time course protocol for PMN depletion, bacterial inoculation, spleen bacterial counts, histopathological examination and analysis of immune cells is presented in Figure S1.

### Ethics statement

Animal experimentation was conducted in strict accordance with good animal practice as defined by the French animal welfare bodies (Law 87–848 dated 19 October 1987 modified by Decree 2001-464 and Decree 2001-131 relative to European Convention, EEC Directive 86/609). All animal work was approved by the Direction Départmentale des Services Vétérinaires des Bouches du Rhônes (authorization number 13.118). All animals were handled and sacrificed according to the approval and guidelines established by the “Comité Institucional para el cuido y uso de los animales” of the Universidad Nacional, and in agreement with the corresponding law “Ley de Bienestar de los Animales No 7451” of Costa Rica (http://www.micit.go.cr/index.php/docman/doc_details/101-ley-no-7451-leydebienestar-de-los-animales.html).

### Antibodies

APC anti-CD5 (53-7.3), PE anti-CD45R/B220 (RA3-6B2), PE-Cy7 anti-CD4 (RM4–5), PB anti-CD8α (53-6.7), FITC anti-CD44 (IM7), PerCP-Cy5.5 anti-CD45.2 (104), PB anti-CD11b (M1/70), FITC anti-Ly6C (AL-21), PE anti- Ly6G (1A8), PE-Cy7 anti-CD11c (HL3), FITC anti-NK1.1 (PK136) antibodies were purchased from BD Biosciences, Alexa Fluor 700 anti-MHC II (M5/114.15.2) from eBiosciense and of RB6-8C5 neutralizing antibody from Bio X cell.

### Bacterial counts and cell analysis

Lymph nodes (axillary, popliteal and mesenteric), blood and spleens were collected at the indicated times after infection (Figure S1), following standard procedures [18]. Spleens were cut in half and each part weighed before processed. The first half was homogenized in 1 mL sterile PBS, and bacterial counts estimated as described elsewhere [22], [30]. The second half of the spleen was processed for population cell estimation by flow cytometry [18]. Briefly, tissues were first cut in small pieces and incubated with a mixture of 500 µL of RPMI containing type II collagenase (Gibco) and of DNAse (Sigma-Aldrich) for 30 min at 37°C in a water bath and re-suspending every 10 minutes to obtain a homogeneous cell suspension. Cell suspensions were filtered in 70 µm membrane Cell Strainers (BD), washed once with RPMI 2% FCS, centrifuged and re-suspended in FACS buffer (PBS 1×, EDTA 2 mM, FCS 2%) prior staining. Blood was collected in EDTA tubes and used directly for antibody staining.

### Flow cytometry and histopathology

Before staining with different antibody mixes, isolated cells were pre-incubated on ice for at least 20 min with the 2.4 G2 mAb to block Fc receptors. Multiparameter FACS analysis was performed using a FACSCanto system (BD Biosciences). FACS data were analyzed using FlowJo software (Tree Star, Inc.). For each experiment, control mice were included to define the proper gates. Antibodies used for identifying each cell population are indicated in Figure S7. Dead cell populations were removed from analysis with LIVE/DEAD Fixable Aqua Dead Cell Stain (Molecular Probes) using AmCyan (Canto) channel. Blood was stained directly with the antibodies and lysed with BD lysing buffer (BD, Biosciences). All samples were fixed in paraphormaldehyde 3.2% after staining and diluted in PBS prior acquisition. For histopatologycal studies, spleens from infected and PBS-treated mice were fixed in 10% neutral buffered formalin, processed and stained with hematoxylin and eosin or Giemsa stain as described elsewhere [54]. In some cases spleen sections were subjected to *Brucella* antigen detection as described previously [55].

### Cytokine measurement

The levels of IL-2, IL-4, IL-6, IFN-γ, TNF-α, IL-17a, IL-10 were measured by CBA Mouse Th1/Th2/Th17 Cytokine Kit (BD, Biosciences) according to the manufacturer's specifications in sera of PMN-depleted, Genista and WT *B. abortus* infected mice. The data were analyzed using FCAP Array software from BD. A pool of normal sera from C57BL/6 mice was used for background estimation.

## Supporting Information

Figure S1 **Experimental design.** Top arrows indicate the days at which a group of mice were treated with anti-RB6-8C5 for PMN depletion. Bottom arrows indicate the days of i.p. infection with 10^6^ CFUs *B. abortus* 2308, determination of spleen bacterial counts, histopathology and flow cytometry analysis (spleen, lymph nodes and blood cells), respectively. C57BL/6 (WT), C57BL/6-PMN mutant (Genista) and C57BL/6 PMN-depleted (PMN-depleted) mice. PMNs basal level over time after treatment with anti-RB6-8C5 is shown with a green dashed line and demonstrated by flow cytometry (A–C) at the indicated times. Course of brucellosis according to Grilló et al. [32] onset of infection (red marks), acute phase (blue marks) chronic phase (yellow marks).
(TIF)

Figure S2 **Bacterial initial spleen colonization in WT and PMN-depleted mice.** (A) CFU/spleen and (B) spleen weights were determined at 16 h in WT and PMN-depleted C57BL/6 mice after i.p. infection with 10^6^ CFUs of *B. abortus* 2308.
(TIF)

Figure S3 **Bacterial loads, spleen weights and cytokine levels detected in WT and Genista mice at the chronic phase of infection (21 days post-infection).** (A) CFU/spleen, (B) spleen weights and (C) levels of INF-γ, IL-6, TNF-α and IL-10 were determined after 21 days in C57BL/6 WT and Genista mice i.p. infected with 10^6^ CFUs of *B. abortus* 2308. Background levels of cytokines obtained in PBS injected mice were subtracted from the values from *Brucella* infected mice. Values of p<0.01 (**) were determined in relation to WT infected mice.
(TIF)

Figure S4 **Lymphoid depletion, macrophage infiltration and granuloma formation becomes more prominent in spleens of PMN-depleted and Genista than in WT** ***Brucella*** **infected mice.** Spleens from infected (1×10^6^ CFUs) and PBS-treated mice were fixed and stained with hematoxylin and eosin stain. For comparison purposes, the pictures in the bottom panel at 100× correspond to the sections indicated by punctuated rectangles in the pictures of the upper panel (40×) already depicted in Figure 3. The insert in the WT bottom panel demonstrate extramedullary hematopoiesis. Top panel 40×, of the same section. Notice the prominent granulomas in the PMN depleted mice (white arrow), while in Genista mice, granulomas are more abundant and most of them have fused (yellow arrows). White pulp (WP) and red pulp (RP).
(TIF)

Figure S5 **Bacterial loads, spleen weights and level of INF-γ detected in WT mice after infection with different bacterial doses.** WT mice were i.p. infected with increasing amounts of *B. abortus* 2308 ranging from 1×10^3^ to 1×10^7^ CFUs. After 5 days of infection, (A) levels of INF-γ and CFU/spleen in relation to bacterial doses, and (B) spleen weights in relations to CFU/spleen were determined. Values of p<0.05 (*), p<0.01 (**) in relation to the lower bacterial dose (10^3^ CFU) are indicated.
(TIF)

Figure S6 ***Brucella abortus*** **barely induces IL-2, IL-4 and IL-17a cytokines in WT and PMN-deficient mice over time.** The levels of cytokines were determined in the sera of C57BL/6 WT, PMN-depleted and Genista mice i.p infected with 10^6^ CFUs of *B. abortus* 2308 at 5, 8 and 15 days post-infection. Background cytokine levels of PBS injected mice (dashed line) are depicted.
(TIF)

Figure S7 **Flow chart depicting the gating strategy for flow cytometry analysis.** Cells isolated from lymph nodes, spleen or blood were analyzed by flow cytometry using various antibody mixes for discriminating against the required cell markers. Boxes indicate the enriched gated populations. (Mo) monocytes, (Gr) granulocytes, (DCs) dendritic cells and, (PMNs) neutrophils, (NK) natural killer cells.
(TIF)

Table S1 **Leucocytes in spleen from infected and non-infected WT, PMN-depleted and Genista mice.** Cells were analyzed by flow cytometry at 8 and 15 days of infection using CD4+/CD44+, CD8+/CD44+, B220+/CD95+, and CD11b+/Ly6C+ cell markers. The percentages of cells found in each of the specified gates are indicated.
(DOCX)

Table S2 **Leucocytes in blood from infected and non-infected WT, PMN-depleted and Genista mice.** Cells were analyzed by flow cytometry at 8 and 15 days of infection using CD4+/CD44+, CD8+/CD44+, B220+/CD95+, and CD11b+/Ly6C+ cell markers. The percentages of cells found in each of the specified gates are indicated.
(DOCX)
