# Supplementary figures and images for: Neutrophils Exert a Suppressive Effect on Th1 Responses to Intracellular Pathogen Brucella abortus
Source: PLoS Pathog. 2013 Feb 14;9(2):e1003167. doi: 10.1371/journal.ppat.1003167 (PMC3573106; doi:10.1371/journal.ppat.1003167)

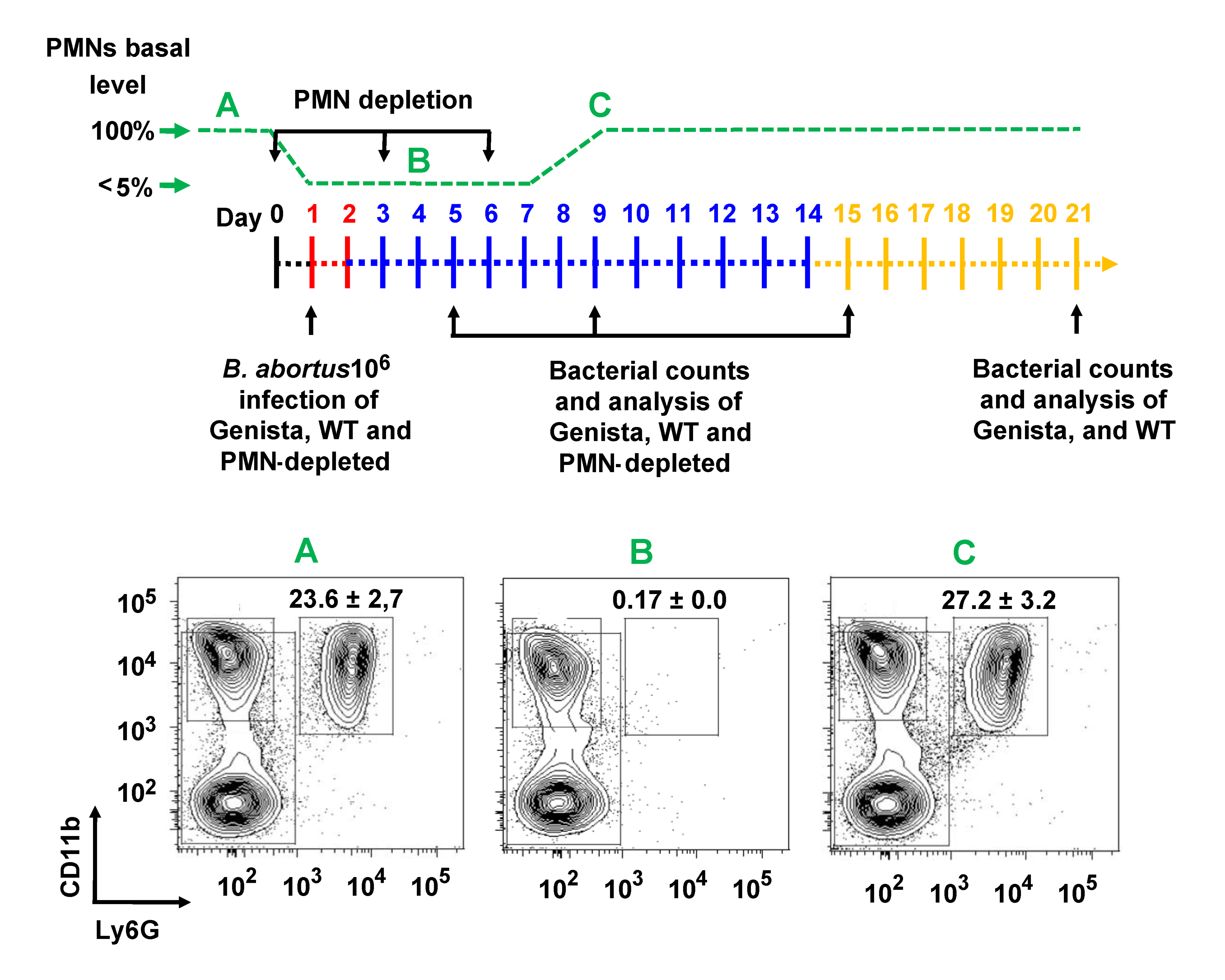

Supplement: Figure S1 — Experimental design. Top arrows indicate the days at which a group of mice were treated with anti-RB6-8C5 for PMN depletion. Bottom arrows indicate the days of i.p. infection with 106 CFUs B. abortus 2308, determination of spleen bacterial counts, histopathology and flow cytometry analysis (spleen, lymph nodes and blood cells), respectively. C57BL/6 (WT), C57BL/6-PMN mutant (Genista) and C57BL/6 PMN-depleted (PMN-depleted) mice. PMNs basal level over time after treatment with anti-RB6-8C5 is shown with a green dashed line and demonstrated by flow cytometry (A–C) at the indicated times. Course of brucellosis according to Grilló et al. [32] onset of infection (red marks), acute phase (blue marks) chronic phase (yellow marks). (TIF) [file ppat.1003167.s001.tif]

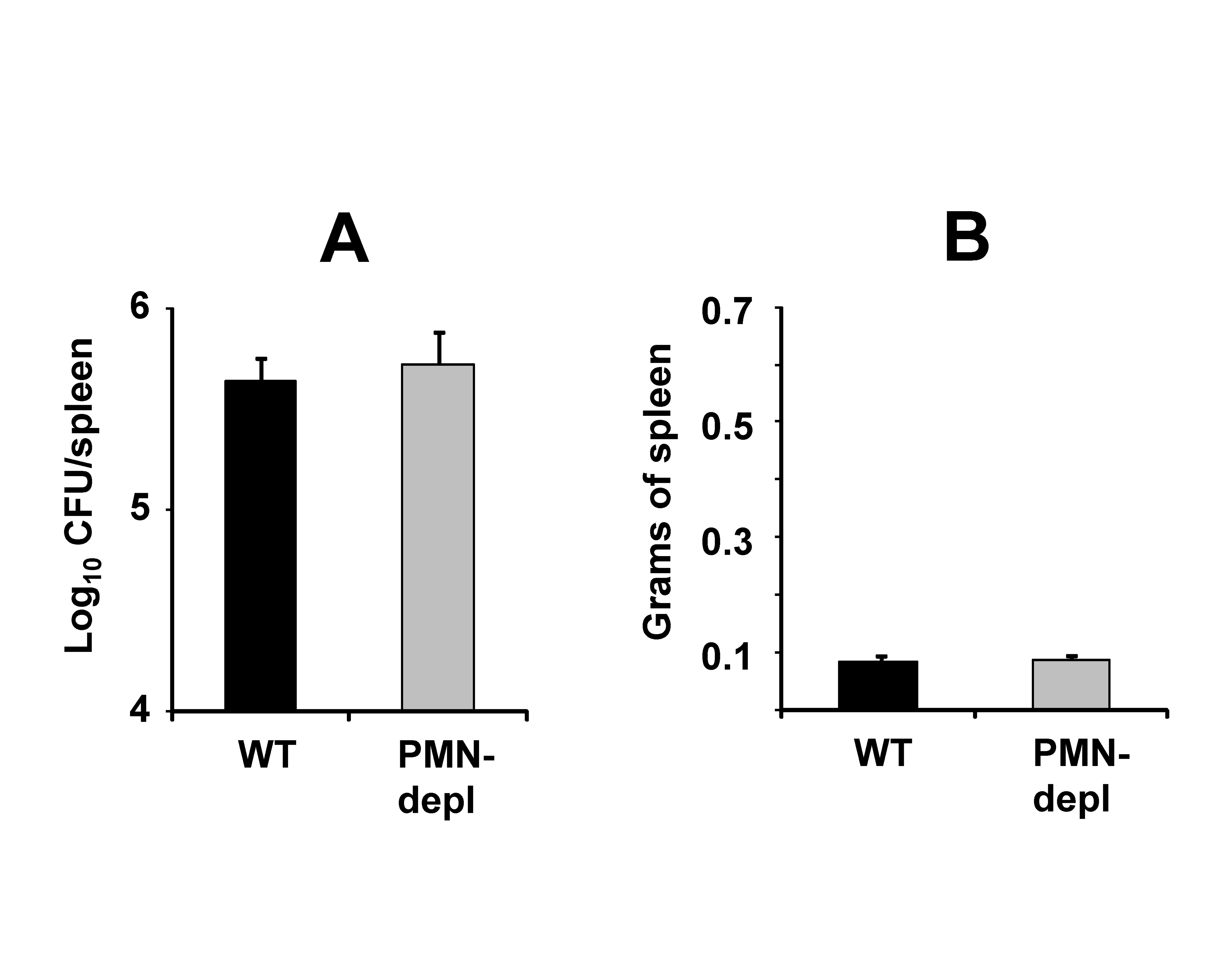

Supplement: Figure S2 — Bacterial initial spleen colonization in WT and PMN-depleted mice. (A) CFU/spleen and (B) spleen weights were determined at 16 h in WT and PMN-depleted C57BL/6 mice after i.p. infection with 106 CFUs of B. abortus 2308. (TIF) [file ppat.1003167.s002.tif]

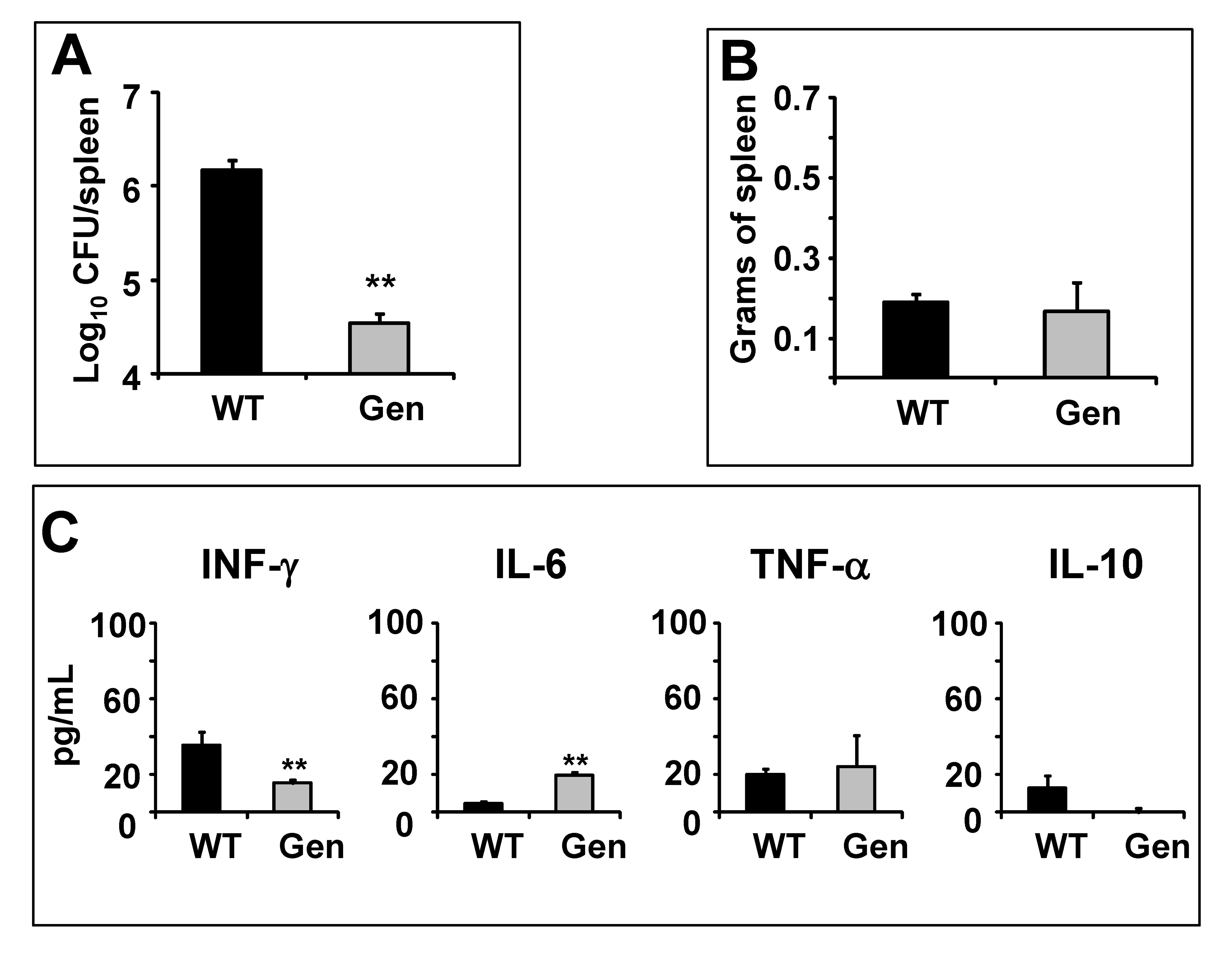

Supplement: Figure S3 — Bacterial loads, spleen weights and cytokine levels detected in WT and Genista mice at the chronic phase of infection (21 days post-infection). (A) CFU/spleen, (B) spleen weights and (C) levels of INF-γ, IL-6, TNF-α and IL-10 were determined after 21 days in C57BL/6 WT and Genista mice i.p. infected with 106 CFUs of B. abortus 2308. Background levels of cytokines obtained in PBS injected mice were subtracted from the values from Brucella infected mice. Values of p<0.01 (**) were determined in relation to WT infected mice. (TIF) [file ppat.1003167.s003.tif]

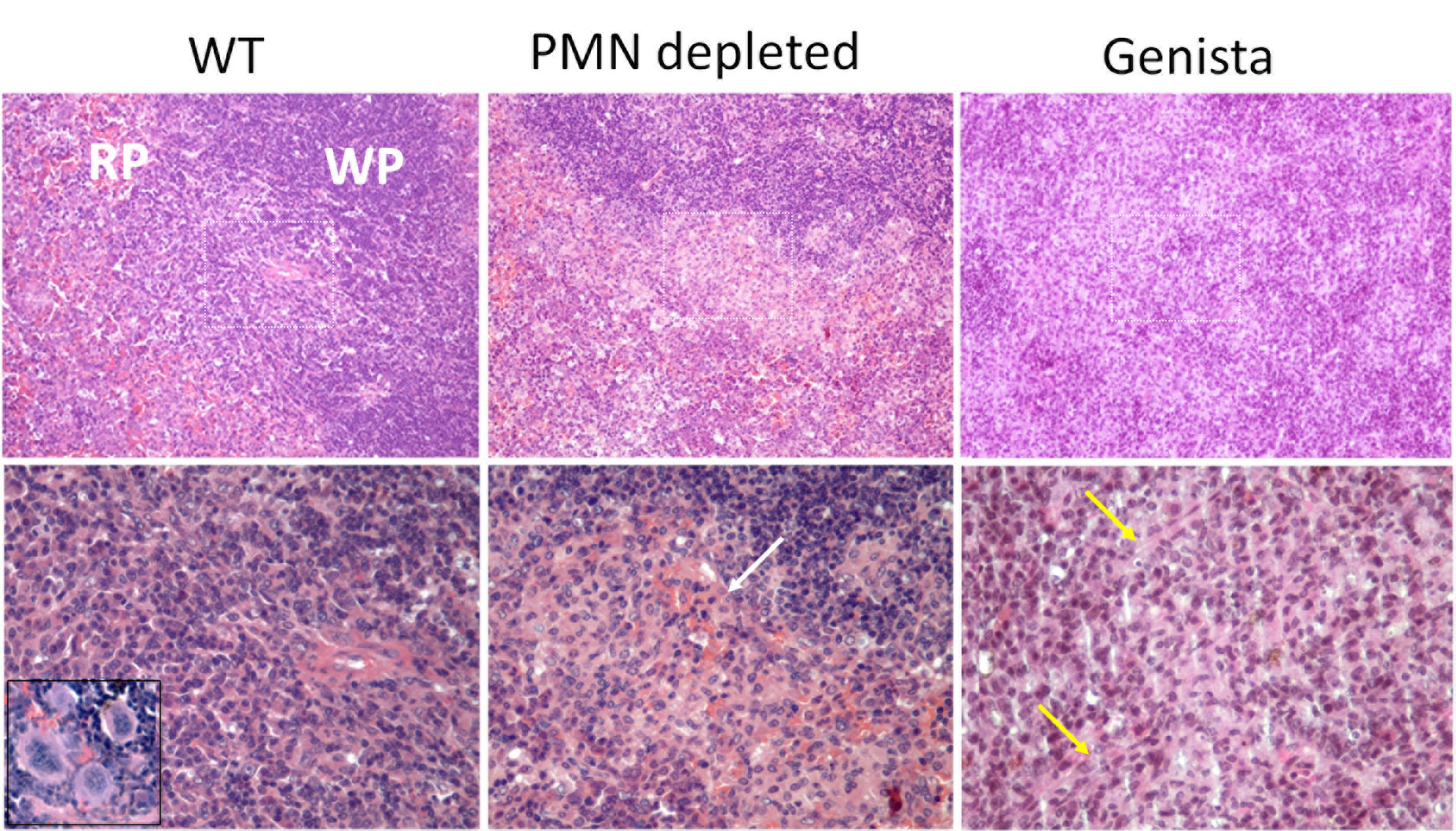

Supplement: Figure S4 — Lymphoid depletion, macrophage infiltration and granuloma formation becomes more prominent in spleens of PMN-depleted and Genista than in WT Brucella infected mice. Spleens from infected (1×10^6^ CFUs) and PBS-treated mice were fixed and stained with hematoxylin and eosin stain. For comparison purposes, the pictures in the bottom panel at 100× correspond to the sections indicated by punctuated rectangles in the pictures of the upper panel (40×) already depicted in Figure 3. The insert in the WT bottom panel demonstrate extramedullary hematopoiesis. Top panel 40×, of the same section. Notice the prominent granulomas in the PMN depleted mice (white arrow), while in Genista mice, granulomas are more abundant and most of them have fused (yellow arrows). White pulp (WP) and red pulp (RP). (TIF) [file ppat.1003167.s004.tif]

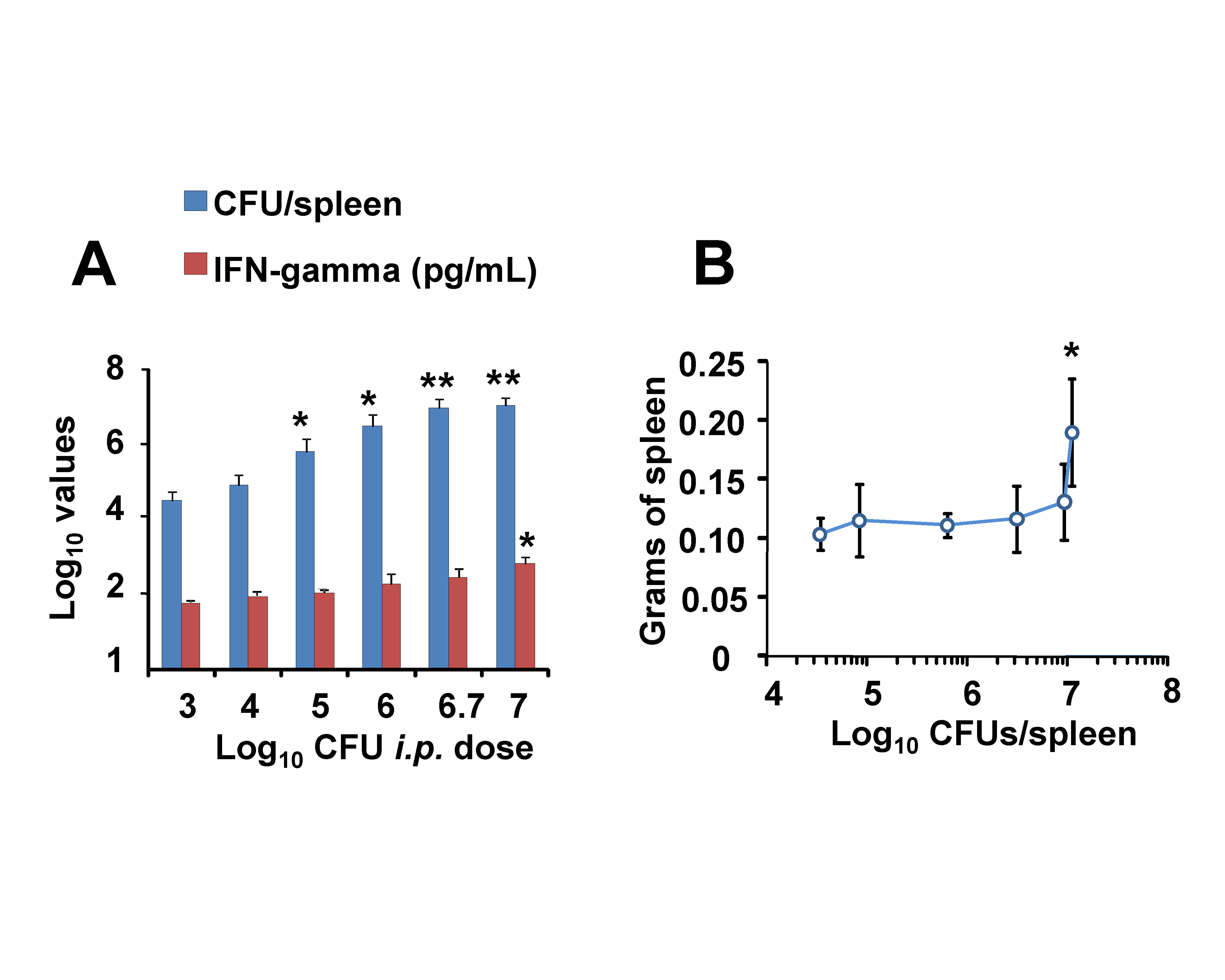

Supplement: Figure S5 — Bacterial loads, spleen weights and level of INF-γ detected in WT mice after infection with different bacterial doses. WT mice were i.p. infected with increasing amounts of B. abortus 2308 ranging from 1×10^3^ to 1×10^7^ CFUs. After 5 days of infection, (A) levels of INF-γ and CFU/spleen in relation to bacterial doses, and (B) spleen weights in relations to CFU/spleen were determined. Values of p<0.05 (*), p<0.01 (**) in relation to the lower bacterial dose (103 CFU) are indicated. (TIF) [file ppat.1003167.s005.tif]

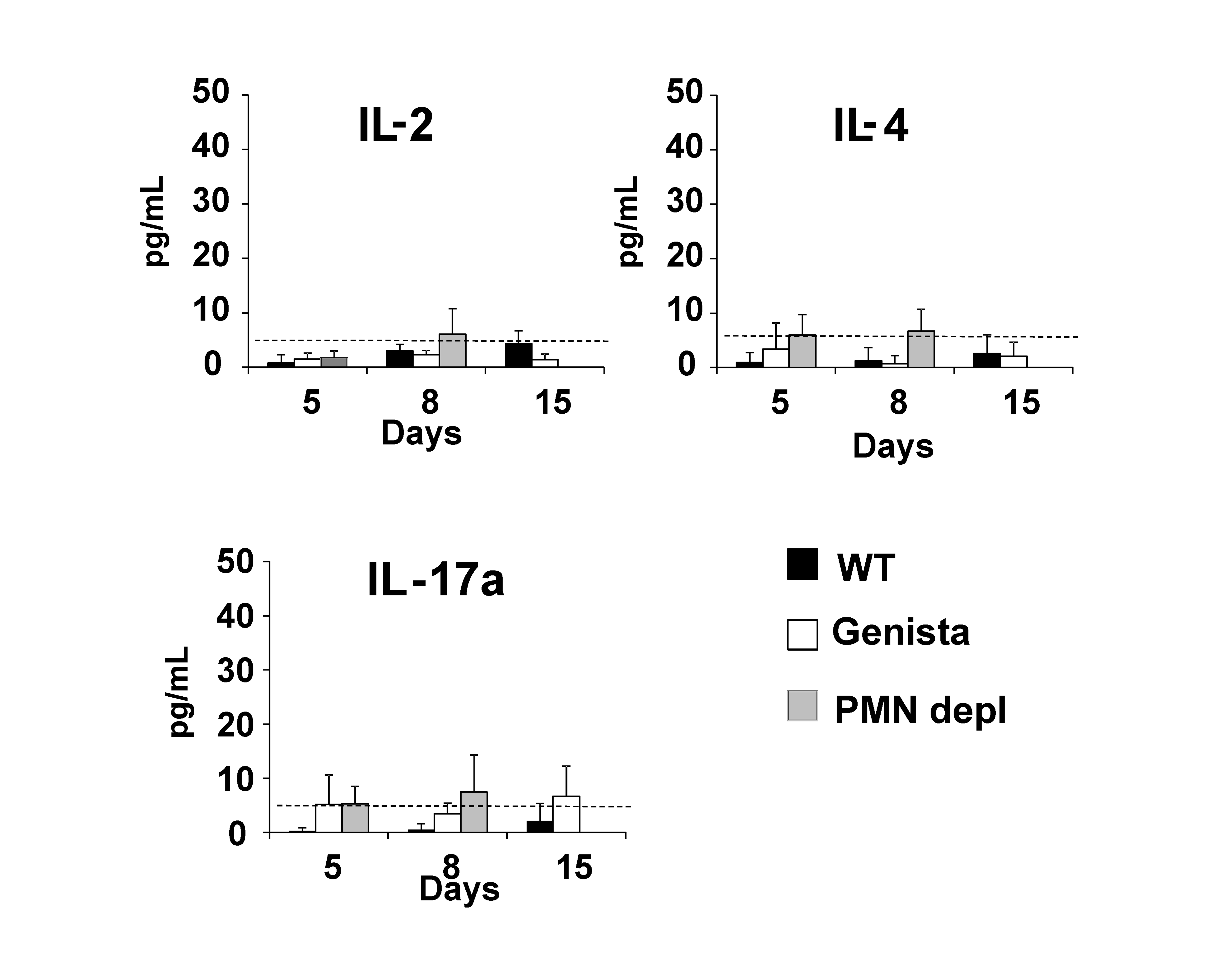

Supplement: Figure S6 — Brucella abortus barely induces IL-2, IL-4 and IL-17a cytokines in WT and PMN-deficient mice over time. The levels of cytokines were determined in the sera of C57BL/6 WT, PMN-depleted and Genista mice i.p infected with 106 CFUs of B. abortus 2308 at 5, 8 and 15 days post-infection. Background cytokine levels of PBS injected mice (dashed line) are depicted. (TIF) [file ppat.1003167.s006.tif]

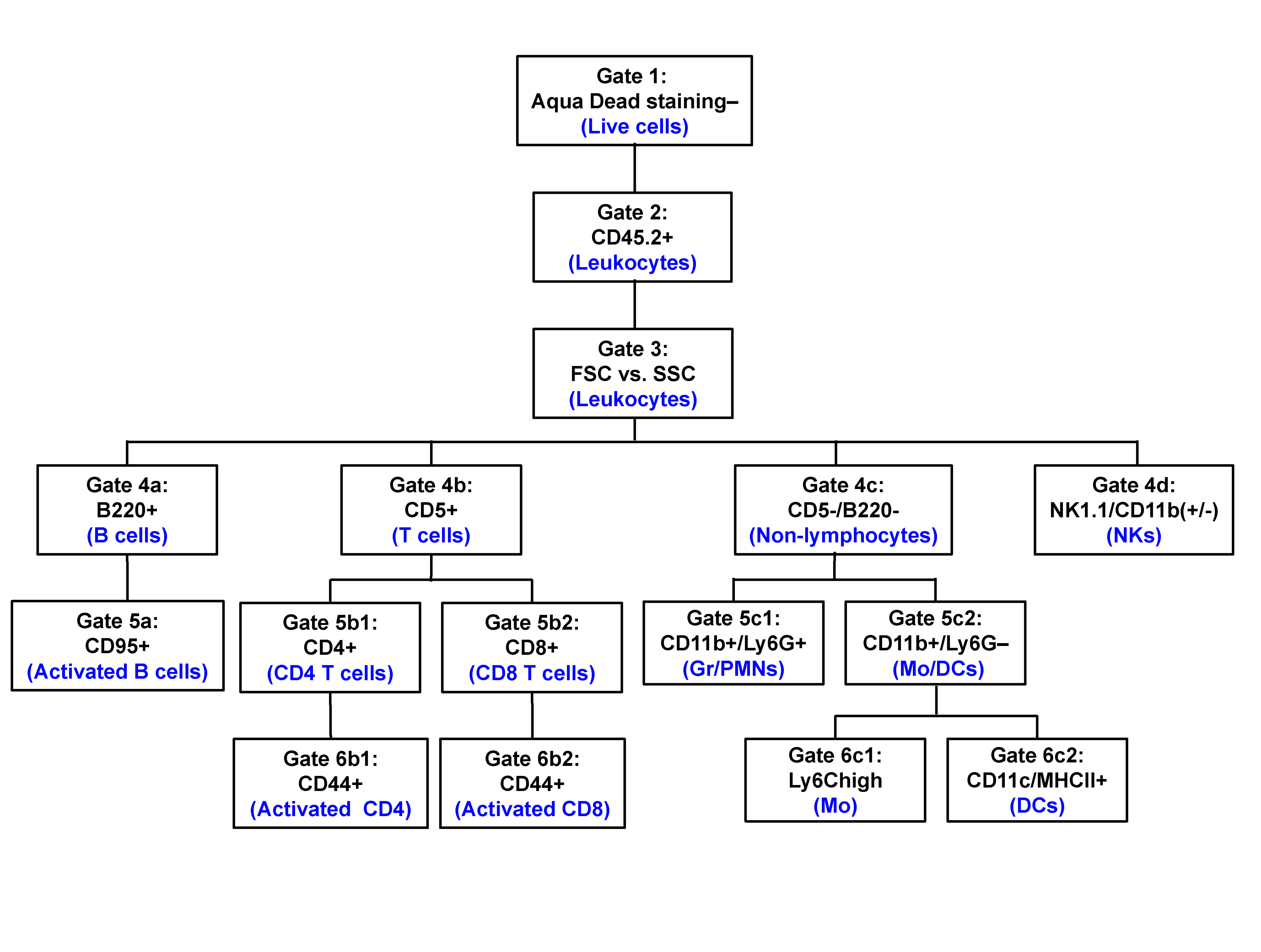

Supplement: Figure S7 — Flow chart depicting the gating strategy for flow cytometry analysis. Cells isolated from lymph nodes, spleen or blood were analyzed by flow cytometry using various antibody mixes for discriminating against the required cell markers. Boxes indicate the enriched gated populations. (Mo) monocytes, (Gr) granulocytes, (DCs) dendritic cells and, (PMNs) neutrophils, (NK) natural killer cells. (TIF) [file ppat.1003167.s007.tif]
