# Supplementary material for: Neutrophils Exert a Suppressive Effect on Th1 Responses to Intracellular Pathogen Brucella abortus
Source: PLoS Pathog. 2013 Feb 14;9(2):e1003167. doi: 10.1371/journal.ppat.1003167 (PMC3573106; doi:10.1371/journal.ppat.1003167)
Supplement: Table S1 — Leucocytes in spleen from infected and non-infected WT, PMN-depleted and Genista mice. Cells were analyzed by flow cytometry at 8 and 15 days of infection using CD4+/CD44+, CD8+/CD44+, B220+/CD95+, and CD11b+/Ly6C+ cell markers. The percentages of cells found in each of the specified gates are indicated. (DOCX) [file ppat.1003167.s008.docx]

**Table S1.** Leucocytes in spleen from infected and non-infected WT, PMN-depleted and Genista mice. Cells were analyzed by flow cytometry at 8 and 15 days of infection using CD4+/CD44+, CD8+/CD44+, B220+/CD95+, and CD11b+/Ly6C+ cell markers. The percentages of cells found in each of the specified gates are indicated.

|  |  |  |  | **Mice** | | | | | | | | | | |
| --- | --- | --- | --- | --- | --- | --- | --- | --- | --- | --- | --- | --- | --- | --- |
|  |  |  |  |  |  |  |  |  |  |  |  |  |  |  |
| **Time** |  | **Cell markers** |  | **Wild type** | | |  | **Genista** | | |  | **PMN-depleted** | | |
|  |  |  |  |  |  |  |  |  |  |  |  |  |  |  |
|  |  |  |  | **Non-Infected** |  | **Infected** |  | **Non-Infected** |  | **Infected** |  | **Non-Infected** |  | **Infected** |
|  |  |  |  |  |  |  |  |  |  |  |  |  |  |  |
| **8 days** |  | CD4+/CD44+ |  | 37.3 ± 5.7 |  | 65.4 ± 5.8 |  | 59.3 ± 3.0 |  | 75.2 ± 5.7 |  | 41.3 ± 1.9 |  | 76.0 ± 7.4 |
|  |  |  |  |  |  |  |  |  |  |  |  |  |  |  |
|  |  | CD8+/CD44+ |  | 40.3 ± 2.5 |  | 35.7 ± 4.5 |  | 81.0 ± 3.5 |  | 69.4 ± 4.5 |  | 35.2 ±3.3 |  | 49.0 ± 6.0 |
|  |  |  |  |  |  |  |  |  |  |  |  |  |  |  |
|  |  | B220+/CD95+ |  | 0.6 ± 0.2 |  | 3.2 ± 0.7 |  | 2.1 ± 1.1 |  | 3.5 ± 1.0 |  | 0.8 ± 0.1 |  | 3.0 ± 0.6 |
|  |  |  |  |  |  |  |  |  |  |  |  |  |  |  |
|  |  | CD11b+/Ly6C+ |  | 12.1 ± 1.1 |  | 49.4 ± 7.3 |  | 15.6 ± 5.3 |  | 77.2 ± 10.0 |  | 33.0 ± 8.5 |  | 80.0 ± 6.8 |
|  |  |  |  |  |  |  |  |  |  |  |  |  |  |  |
|  |  |  |  |  |  |  |  |  |  |  |  |  |  |  |
| **15 days** |  | CD4+/CD44+ |  | 19.8 ± 4.4 |  | ND |  | 41.6 ± 6.1 |  | 69.2 ± 8.8 |  |  |  |  |
|  |  |  |  |  |  |  |  |  |  |  |  |  |  |  |
|  |  | CD8+/CD44+ |  | 28.1 ± 3.9 |  | ND |  | 64.8 ± 5.3 |  | 82.3 ± 6.5 |  |  |  |  |
|  |  |  |  |  |  |  |  |  |  |  |  |  |  |  |
|  |  | B220+/CD95+ |  | 1.2 ± 0.2 |  | ND |  | 3.4 ± 1.3 |  | 6.0 ± 3.8 |  |  |  |  |
|  |  |  |  |  |  |  |  |  |  |  |  |  |  |  |
|  |  | CD11b+/Ly6C+ |  | 15.4 ± 1.8 |  | 13.3 ± 0.7 |  | 13.0 ± 2.1 |  | 66.0 ± 12.5 |  |  |  |  |
