# Supplementary material for: Neutrophils Exert a Suppressive Effect on Th1 Responses to Intracellular Pathogen Brucella abortus
Source: PLoS Pathog. 2013 Feb 14;9(2):e1003167. doi: 10.1371/journal.ppat.1003167 (PMC3573106; doi:10.1371/journal.ppat.1003167)
Supplement: Table S2 — Leucocytes in blood from infected and non-infected WT, PMN-depleted and Genista mice. Cells were analyzed by flow cytometry at 8 and 15 days of infection using CD4+/CD44+, CD8+/CD44+, B220+/CD95+, and CD11b+/Ly6C+ cell markers. The percentages of cells found in each of the specified gates are indicated. (DOCX) [file ppat.1003167.s009.docx]

**Table S2.** Leucocytes in blood from infected and non-infected WT, PMN-depleted and Genista mice. Cells were analyzed by flow cytometry at 8 and 15 days of infection using CD4+/CD44+, CD8+/CD44+, B220+/CD95+, and CD11b+/Ly6C+ cell markers. The percentages of cells found in each of the specified gates are indicated.

|  |  |  |  | **Mice** | | | | | | | | | | |
| --- | --- | --- | --- | --- | --- | --- | --- | --- | --- | --- | --- | --- | --- | --- |
|  |  |  |  |  |  |  |  |  |  |  |  |  |  |  |
| **Time** |  | **Cell markers** |  | **Wild type** | | |  | **Genista** | | |  | **PMN-depleted** | | |
|  |  |  |  |  |  |  |  |  |  |  |  |  |  |  |
|  |  |  |  | **Non-Infected** |  | **Infected** |  | **Non-Infected** |  | **Infected** |  | **Non-Infected** |  | **Infected** |
|  |  |  |  |  |  |  |  |  |  |  |  |  |  |  |
| **8 days** |  | CD4+/CD44+ |  | 16.2 ± 2.7 |  | 79.3 ± 4.3 |  | 35.3 ± 4.2 |  | 81.9 ± 1.7 |  | 16.3 ± 2.6 |  | 85.6 ± 12.4 |
|  |  |  |  |  |  |  |  |  |  |  |  |  |  |  |
|  |  | CD8+/CD44+ |  | 28.9 ± 2.5 |  | 47.8 ± 8.0 |  | 81.7 ± 0.7 |  | 78.3 ± 2.0 |  | 20.3 ± 4.2 |  | 89.9 ± 9.9 |
|  |  |  |  |  |  |  |  |  |  |  |  |  |  |  |
|  |  | B220+/CD95+ |  | 1.1 ± 0.4 |  | 1.0 ± 0.6 |  | 1.4 ± 0.4 |  | 3.4 ± 1.7 |  | 1.9 ± 1.3 |  | 2.6 ± 0.2 |
|  |  |  |  |  |  |  |  |  |  |  |  |  |  |  |
|  |  | CD11b+/Ly6C+ |  | 49.9 ± 5.1 |  | 91.5 ± 2.5 |  | 55.7 ± 5.3 |  | 91.2 ± 2.5 |  | 29.1 ± 1.3 |  | 77.3 ± 4.3 |
|  |  |  |  |  |  |  |  |  |  |  |  |  |  |  |
|  |  |  |  |  |  |  |  |  |  |  |  |  |  |  |
| **15 days** |  | CD4+/CD44+ |  | 7.1 ± 0.4 |  | 62.6 ± 2.2 |  | 11.1 ± 2.0 |  | 76.2 ± 7.7 |  |  |  |  |
|  |  |  |  |  |  |  |  |  |  |  |  |  |  |  |
|  |  | CD8+/CD44+ |  | 17.5 ± 1.7 |  | 82.4 ± 4.0 |  | 48.6 ± 6.4 |  | 93.7 ± 3.6 |  |  |  |  |
|  |  |  |  |  |  |  |  |  |  |  |  |  |  |  |
|  |  | B220+/CD95+ |  | 1.4 ± 0.6 |  | 1.9 ± 0.6 |  | 1.2 ± 0.5 |  | 2.3 ± 0.3 |  |  |  |  |
|  |  |  |  |  |  |  |  |  |  |  |  |  |  |  |
|  |  | CD11b+/Ly6C+ |  | 37.2 ± 5.2 |  | 94.3 ± 1.3 |  | 58.2 ± 4.9 |  | 84.7 ± 9.7 |  |  |  |  |
